# Supplementary material for: The association between serum lipids and risk of premature mortality in Latin America: a systematic review of population-based prospective cohort studies
Source: PeerJ. 2019 Oct 4;7:e7856. doi: 10.7717/peerj.7856 (PMC6779113; doi:10.7717/peerj.7856)
Supplement: Supplemental Information 2 [file peerj-07-7856-s002.docx]

**Rational**

Epidemiological studies have reported inconsistent findings about the association between lipids and premature cardiovascular mortality.(Di Angelantonio et al. 2009; Lewington et al. 2007) The Prospective Studies Collaboration reported a lower hazard of death due to ischaemic heart disease for each 1 mmol/L reduction of total cholesterol.(Lewington et al. 2007) However, the evidence of an association between serum lipids and cerebrovascular disease mortality was less consistent in their study.(Lewington et al. 2007) Furthermore, the Emerging Risk Factor Collaboration revealed a higher hazard of death due to coronary heart diseases for people with increased cholesterol and LDL-cholesterol levels and those with decreased HDL-cholesterol.(Di Angelantonio et al. 2009) Again, the evidence was less conclusive when the main outcome was ischaemic stroke mortality.(Di Angelantonio et al. 2009) Other independent prospective studies have reported that the association between total cholesterol and stroke mortality varies according to stroke sub-type.(Yi et al. 2018) Moreover, a study including people aged 60 years and above reported a reduced mortality between increased serum total cholesterol and all-cause mortality, most likely due to a high number of non-cardiovascular deaths;(Liang et al. 2017) similarly, LDL-cholesterol seems to have an negative correlation with mortality in people 60 years old and above.(Ravnskov et al. 2016) Whether these observations hold in populations with different distribution of cholesterol, health profiles and access to healthcare,(Atun et al. 2015; Cotlear et al. 2015; Farzadfar et al. 2011) deserves verification.

It must be kept in mind that the current scientific evidence on the association between serum lipids and mortality is derived mostly from Western populations. Therefore, summarizing studies on the association between lipid biomarkers and mortality in LA populations may provide valuable information for the development of local guidelines for clinicians and health policy makers. The objective of this study was to synthetize, through a systematic review, the current scientific evidence on the association between serum lipids and premature mortality in LA.

**Contribution**

This systematic review of the literature in LA did not reveal scientific evidence on an association between unfavourable serum lipid biomarkers and premature mortality in the general population. Furthermore, the definitions used to categorize lipid biomarkers were inconsistent across reports. In addition, only one study was conducted within the last ten years. Overall, our findings call to either conduct new cohort studies or use available ones to systematically estimate the mortality risk associated with lipid profiles, using consistent metrics and clinically relevant definitions. Thus, there is a need to study the long-term effects of lipid profiles as this will provide evidence to inform local clinical practice, health policy and priority setting for LA.

To date, it is not possible to ascertain the association between lipid biomarkers and mortality risk in LA. The available evidence is outdated, and the definitions of lipid biomarkers are inconsistent. In addition, different methods were used to measure the long-term mortality risk in LA populations. These findings strongly suggest conducting larger studies within the LA population to get valuable risk estimates of the associations between serum lipids and premature mortality

Atun R, de Andrade LO, Almeida G, Cotlear D, Dmytraczenko T, Frenz P, Garcia P, Gomez-Dantes O, Knaul FM, Muntaner C, de Paula JB, Rigoli F, Serrate PC, and Wagstaff A. 2015. Health-system reform and universal health coverage in Latin America. *Lancet* 385:1230-1247. 10.1016/s0140-6736(14)61646-9

Cotlear D, Gomez-Dantes O, Knaul F, Atun R, Barreto IC, Cetrangolo O, Cueto M, Francke P, Frenz P, Guerrero R, Lozano R, Marten R, and Saenz R. 2015. Overcoming social segregation in health care in Latin America. *Lancet* 385:1248-1259. 10.1016/s0140-6736(14)61647-0

Di Angelantonio E, Sarwar N, Perry P, Kaptoge S, Ray KK, Thompson A, Wood AM, Lewington S, Sattar N, Packard CJ, Collins R, Thompson SG, and Danesh J. 2009. Major lipids, apolipoproteins, and risk of vascular disease. *JAMA* 302:1993-2000. 10.1001/jama.2009.1619

Farzadfar F, Finucane MM, Danaei G, Pelizzari PM, Cowan MJ, Paciorek CJ, Singh GM, Lin JK, Stevens GA, Riley LM, and Ezzati M. 2011. National, regional, and global trends in serum total cholesterol since 1980: systematic analysis of health examination surveys and epidemiological studies with 321 country-years and 3.0 million participants. *Lancet* 377:578-586. 10.1016/s0140-6736(10)62038-7

Lewington S, Whitlock G, Clarke R, Sherliker P, Emberson J, Halsey J, Qizilbash N, Peto R, and Collins R. 2007. Blood cholesterol and vascular mortality by age, sex, and blood pressure: a meta-analysis of individual data from 61 prospective studies with 55,000 vascular deaths. *Lancet* 370:1829-1839. 10.1016/s0140-6736(07)61778-4

Liang Y, Vetrano DL, and Qiu C. 2017. Serum total cholesterol and risk of cardiovascular and non-cardiovascular mortality in old age: a population-based study. *BMC Geriatr* 17:294. 10.1186/s12877-017-0685-z

Ravnskov U, Diamond DM, Hama R, Hamazaki T, Hammarskjold B, Hynes N, Kendrick M, Langsjoen PH, Malhotra A, Mascitelli L, McCully KS, Ogushi Y, Okuyama H, Rosch PJ, Schersten T, Sultan S, and Sundberg R. 2016. Lack of an association or an inverse association between low-density-lipoprotein cholesterol and mortality in the elderly: a systematic review. *BMJ open* 6:e010401. 10.1136/bmjopen-2015-010401

Yi SW, Shin DH, Kim H, Yi JJ, and Ohrr H. 2018. Total cholesterol and stroke mortality in middle-aged and elderly adults: A prospective cohort study. *Atherosclerosis* 270:211-217. 10.1016/j.atherosclerosis.2017.12.003
