# Supplementary material for: The association between serum lipids and risk of premature mortality in Latin America: a systematic review of population-based prospective cohort studies
Source: PeerJ. 2019 Oct 4;7:e7856. doi: 10.7717/peerj.7856 (PMC6779113; doi:10.7717/peerj.7856)
Supplement: Supplemental Information 3 [file peerj-07-7856-s003.docx]

**The association between serum lipids and risk of premature mortality in Latin America: A systematic review of population-based prospective cohort studies**

Rodrigo M Carrillo-Larco

Leonardo Albitres-Flores

Noël C Barengo

Antonio Bernabe-Ortiz

**Corresponding autor**

Rodrigo M Carrillo-Larco, MD

Department of Epidemiology and Biostatistics

School of Public Health, Imperial College London, London W2 1PG, UK

E-mail: [rcarrill@ic.ac.uk](mailto:rcarrill@ic.ac.uk)

Phone: +44 0 7578240395

**Table of Contents**

[Search terms used in Ovid (Embase, Medline, Global Health) 2](#_Toc16107657)

[Search terms used in Scopus 3](#_Toc16107658)

[Search terms used in LILACS 4](#_Toc16107659)

[Risk estimates reported in each study 5](#_Toc16107660)

[Risk of bias 6](#_Toc16107661)

# **Search terms used in Ovid (Embase, Medline, Global Health)**

| 1 | exp Cholesterol/ |
| --- | --- |
| 2 | cholesterol.mp. |
| 3 | 1 or 2 |
|  |  |
| 4 | exp Cholesterol, LDL/ |
| 5 | ldl-cholesterol.mp. |
| 6 | low density lipoprotein cholesterol.mp. |
| 7 | 4 or 5 or 6 |
|  |  |
| 8 | exp Cholesterol, HDL/ |
| 9 | hdl-cholesterol.mp. |
| 10 | high density lipoprotein cholesterol.mp. |
| 11 | 8 or 9 or 10 |
|  |  |
| 12 | exp Triglycerides/ |
| 13 | triglycerides.mp. |
| 14 | 12 or 13 |
|  |  |
| 15 | dyslipidemi$.mp. |
| 16 | lipid disorder.mp. |
| 17 | hypercholesterolemia.mp. |
| 18 | hypertriglyceridemia.mp. |
| 19 | hyperlipidemia.mp. |
| 20 | 15 or 16 or 17 or 18 or 19 |
|  |  |
| 21 | 3 or 7 or 11 or 14 or 20 |
|  |  |
| 22 | (("Antigua and Barbuda") or ("Argentina") or ("Bahamas") or ("Barbados") or ("Belize") or ("Bolivia") or ("Brazil") or ("United States Virgin Islands") or ("British Virgin Islands") or ("Chile") or ("Colombia") or ("Costa Rica") or ("Cuba") or ("Dominica") or ("Dominican Republic") or ("Ecuador") or ("El Salvador") or ("Grenada") or ("Guatemala") or ("Guyana") or ("Haiti") or ("Honduras") or ("Jamaica") or ("Mexico") or ("Nicaragua") or ("Panama") or ("Paraguay") or ("Peru") or ("Puerto Rico") or ("Saint Kitts and Nevis") or ("Saint Lucia") or ("Saint Vincent and the Grenadines") or ("Suriname") or ("Trinidad and Tobago") or ("West Indies") or ("Uruguay") or ("Venezuela") or ("Latin America") or latin amer$ or ("South America") or south amer$ or ("Central America") or central amer$ or ("Caribbean Region")) |
|  |  |
| 23 | mortality.mp. |
| 24 | death.mp. |
| 25 | fatality.mp. |
| 26 | 23 or 24 or 25 |
|  |  |
| 27 | cohort.mp. |
| 28 | follow-up.mp. |
| 29 | followed-up.mp. |
| 30 | panel.mp. |
| 31 | longitudinal.mp. |
| 32 | prospective.mp. |
| 33 | 27 or 28 or 29 or 30 or 31 or 32 |
|  |  |
| 34 | 21 and 22 and 26 and 33 |
| 35 | exp animals/ not humans.sh. |
| 36 | 34 not 35 |
| 37 | remove duplicates from 36 |

# **Search terms used in Scopus**

(TITLE-ABS-KEY(cholesterol) OR TITLE-ABS-KEY(LDL cholesterol) OR TITLE-ABS-KEY(low density lipoprotein cholesterol) OR TITLE-ABS-KEY(HDL cholesterol) OR TITLE-ABS-KEY(high density lipoprotein cholesterol) OR TITLE-ABS-KEY(triglycerides) OR TITLE-ABS-KEY(dyslipidemi*) OR TITLE-ABS-KEY(lipid disorder) OR TITLE-ABS-KEY(hypercholesterolemia) OR TITLE-ABS-KEY(hypertriglyceridemia) OR TITLE-ABS-KEY(hyperlipidemia)) AND (TITLE-ABS-KEY("Antigua and Barbuda") or TITLE-ABS-KEY("Argentina") or TITLE-ABS-KEY("Bahamas") or TITLE-ABS-KEY("Barbados") or TITLE-ABS-KEY("Belize") or TITLE-ABS-KEY("Bolivia") or TITLE-ABS-KEY("Brazil") or TITLE-ABS-KEY("United States Virgin Islands") or TITLE-ABS-KEY("British Virgin Islands") or TITLE-ABS-KEY("Chile") or TITLE-ABS-KEY("Colombia") or TITLE-ABS-KEY("Costa Rica") or TITLE-ABS-KEY("Cuba") or TITLE-ABS-KEY("Dominica") or TITLE-ABS-KEY("Dominican Republic") or TITLE-ABS-KEY("Ecuador") or TITLE-ABS-KEY("El Salvador") or TITLE-ABS-KEY("Grenada") or TITLE-ABS-KEY("Guatemala") or TITLE-ABS-KEY("Guyana") or TITLE-ABS-KEY("Haiti") or TITLE-ABS-KEY("Honduras") or TITLE-ABS-KEY("Jamaica") or TITLE-ABS-KEY("Mexico") or TITLE-ABS-KEY("Nicaragua") or TITLE-ABS-KEY("Panama") or TITLE-ABS-KEY("Paraguay") or TITLE-ABS-KEY("Peru") or TITLE-ABS-KEY("Puerto Rico") or TITLE-ABS-KEY("Saint Kitts and Nevis") or TITLE-ABS-KEY("Saint Lucia") or TITLE-ABS-KEY("Saint Vincent and the Grenadines") or TITLE-ABS-KEY("Suriname") or TITLE-ABS-KEY("Trinidad and Tobago") or TITLE-ABS-KEY("West Indies") or TITLE-ABS-KEY("Uruguay") or TITLE-ABS-KEY("Venezuela") or TITLE-ABS-KEY("Latin America") or TITLE-ABS-KEY(latin amer$) or TITLE-ABS-KEY("South America") or TITLE-ABS-KEY(south amer$) or TITLE-ABS-KEY("Central America") or TITLE-ABS-KEY(central amer$) or TITLE-ABS-KEY("Caribbean Region")) AND (TITLE-ABS-KEY(mortality) OR TITLE-ABS-KEY(death) OR TITLE-ABS-KEY(fatality)) AND (TITLE-ABS-KEY(cohort) OR TITLE-ABS-KEY(follow-up)OR TITLE-ABS-KEY(followed-up) OR TITLE-ABS-KEY(panel) OR TITLE-ABS-KEY(prospective) OR TITLE-ABS-KEY(longitudinal)) NOT DBCOLL(medl) AND (LIMIT-TO ( DOCTYPE , "ar")) AND ( LIMIT-TO(SUBJAREA, "MEDI"))

# **Search terms used in LILACS**

((colesterol) OR (LDL colesterol) OR (lipoproteína de baja densidad) OR (HDL colesterol) OR (lipoproteína de alta densidad) OR (trigliceridos) OR (dislipidemias) OR (hipercolesterolemia) OR (hipertrigliceridemia) OR (hiperlipidemia)) AND(("Antigua y Barbuda") or ("Argentina") or ("Aruba") or ("Bahamas") or ("Barbados") or ("Belice") or ("Bolivia") or ("Brasil") or ("Islas Vírgenes de los Estados Unidos") or ("Islas Vírgenes Británicas") or ("Islas Caimán") or ("Chile") or ("Colombia") or ("Costa Rica") or ("Cuba") or ("Curazao") or ("Dominica") or ("Republica Dominicana") or ("Ecuador") or ("El Salvador") or ("Granada") or ("Guatemala") or ("Guyana") or ("Haití") or ("Honduras") or ("Jamaica") or ("México") or ("Nicaragua") or ("Panamá") or ("Paraguay") or ("Perú") or ("Puerto Rico") or ("San Cristóbal y Nieves ") or ("Santa Lucía") or ("San Vicente y las Granadinas ") or ("Surinam") or ("Trinidad y Tobago") or ("Turcas y Caicos ") or ("Uruguay") or ("Venezuela") or ("América Latina") or ("Latinoamérica") or ("América del Sur") or ("Sudamérica") or ("Suramérica​") or ("América Central") or ("Centroamérica") or ("América del Centro") or ("Caribe")) AND ((mortalidad) OR (muerte) OR (fatalidad)) AND ((cohorte) OR (seguimiento) OR (panel) OR (prospectivo) OR (longitudinal))

**Risk estimates reported in each study**

# **Risk of bias**

| Study | Selection | | | | Comparability | Outcome | | |
| --- | --- | --- | --- | --- | --- | --- | --- | --- |
|  | Representativeness | Selection | Ascertainment | Demonstration | Comparability | Assessment | Long enough | Adequacy |
| Lazo-Porras, 2016 | Truly | Same | Secure | Yes | Controls for additional factors | record | Yes | Small lost, no bias |
| Werle, 2011 | Truly | Same | Secure | No | Controls for additional factors | record | Yes | Small lost, no bias |
| Garcia-Palmieri, 1981 | Truly | Same | Secure | No | Controls for important factors | record | Yes | Small lost, no bias |
| Crespo, 2002 | Truly | Same | Secure | Yes | Controls for additional factors | No description | Yes | Small lost, no bias |
| Garcia-Palmieri, 1988 | Truly | Same | Secure | No | Controls for important factors | record | Yes | Small lost, no bias |

Source: NEWCASTLE - OTTAWA QUALITY ASSESSMENT SCALE COHORT STUDIES

<http://www.ohri.ca/programs/clinical_epidemiology/oxford.asp>
